# Robustness and mode selectivity in parity-time (PT) symmetric lasers

**DOI:** 10.1038/s41598-017-10216-1

**Published:** 2017-09-07

**Authors:** M. H. Teimourpour, M. Khajavikhan, D. N. Christodoulides, R. El-Ganainy

**Affiliations:** 1 Department of Physics and Henes Center for Quantum Phenomena, Michigan Technological University, Houghton, MI 49931 USA; 2 College of Optics & Photonics-CREOL, University of Central Florida, Orlando, Fl 32816 USA

## Abstract

We investigate two important aspects of PT symmetric photonic molecule lasers, namely the robustness of their single longitudinal mode operation against instabilities triggered by spectral hole burning effects, and the possibility of more versatile mode selectivity. Our results, supported by numerically integrating the nonlinear rate equations and performing linear stability analysis, reveals the following: (1) In principle a second threshold exists after which single mode operation becomes unstable, signaling multimode oscillatory dynamics, (2) For a wide range of design parameters, single mode operation of PT lasers having relatively large free spectral range (FSR) can be robust even at higher gain values, (3) PT symmetric photonic molecule lasers are more robust than their counterpart structures made of single microresonators; and (4) Extending the concept of single longitudinal mode operation based on PT symmetry in millimeter long edge emitting lasers having smaller FSR can be challenging due to instabilities induced by nonlinear modal interactions. Finally we also present a possible strategy based on loss engineering to achieve more control over the mode selectivity by suppressing the mode that has the highest gain (i.e. lies under the peak of the gain spectrum curve) and switch the lasing action to another mode.

## Introduction

Semiconductor lasers are indispensable tools that play important roles in several applications such as fiber optics communication networks, optical memory, medical diagnosis and surgery, and sensing. Commercial semiconductor lasers fall into two main categories: surface and edge emitting devices^1–3^, the design details of which can vary widely depending on the material system, photonic architecture, emission wavelength and operation environment. The former can be tailored to support only one longitudinal mode. The latter however, due to the relatively large gain bandwidth curve of semiconductors compared to atomic gas and the longer optical path of a full roundtrip inside the cavity, can be multimoded. Several strategies have been developed to overcome this problem. One common techniques is based on distributed feedback mechanism which relies on periodic modulation of optical refractive index^4^. The quest for continuous miniaturization and lower power consumption (ideally threshold-less devices) has sparked interest in novel laser cavities such as microdiscs^5–12^, microrings^13–15^, photonic crystals^16, 17^ and plasmonics^18^. Although, several methods for achieving single mode emission in these geometries have been proposed^12–14^, a commercially viable option is still lacking.

Recently, the novel concept of PT symmetry^19, 20^ was introduced in optics and photonics^21–25^, which subsequently led to intense investigations^26–43^. Inspired by some of these recent activities, non-Hermitian engineering of laser emission near exceptional points have been theoretically investigated and experimentally reported by several research groups^44–52^. In particular the work in ref. 45 demonstrated the possibility of single mode operation in *PT* symmetric photonic molecules made of microring resonators. Subsequent theoretical studies have investigated nonlinear interactions between the optical supermodes in these systems^53–55^ assuming that each resonator supports only one optical mode (with the exception of ref. 54 which also considered spatial hole burning effects in multimode cavities).

**Figure 1:**
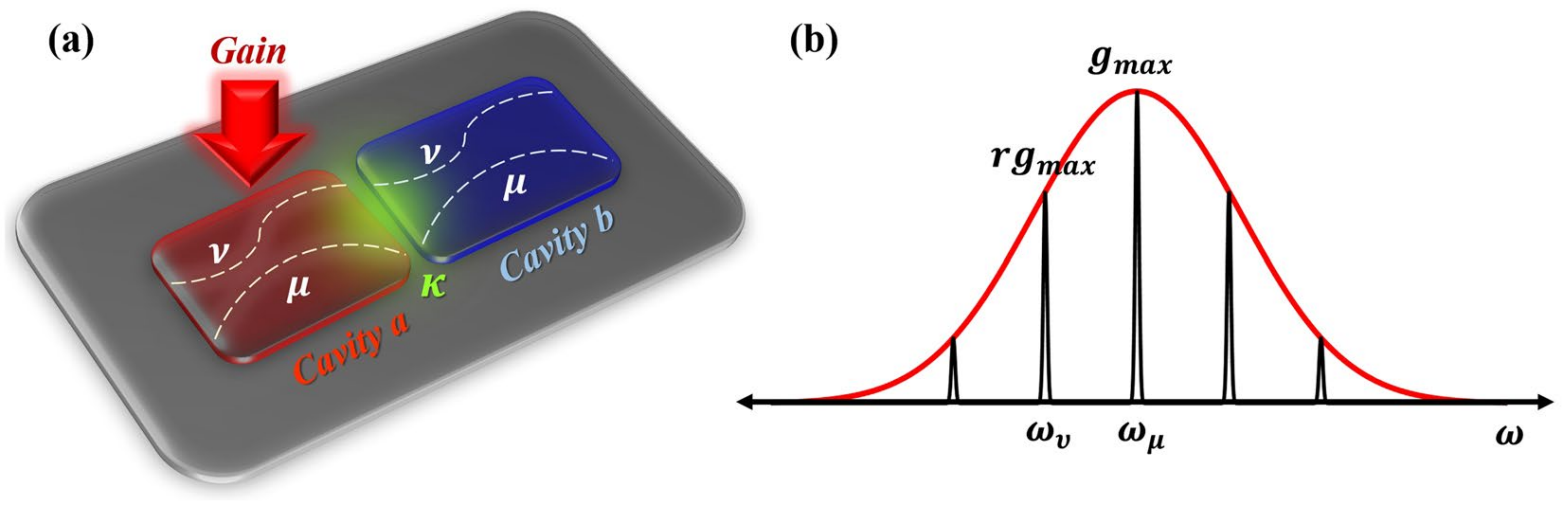
(**a**) A schematic of the photonic molecule laser investigated in this work. It consists of two identical coupled optical cavities (*a* and *b*), each of which supports in general several modes (later we focus only on two modes). The coupling coefficients between each pair of the modes is assumed to be the same and equal *κ*. Moreover, pumping is applied only to cavity *a*. A typical semiconductor gain curve along with the assumed modal frequencies are shown in (**b**) where *g*_*max*_ and *rg*_*max*_ are gain values experienced by modes *μ* and *ν* respectively.

Motivated by these rapid developments, here we investigate PT symmetric photonic molecule lasers (see Fig. 1(a)), taking into consideration the nonlinear interactions between the intrinsic single cavity modes (not just the supermodes). Our study, complementing previous works and providing insight into the operation dynamics of PT symmetric lasers, reveal the following important results: (1) *PT* symmetry can provide superior performance (in terms of single longitudinal mode operation) in microcavities having relatively large FSR, and (2) Extending the concept to millimeter long edge emitting lasers can be challenging due to nonlinear instabilities, and (3) More general loss engineering schemes can be used to achieve more control over mode selectivity.

To this end, we start by considering a photonic molecule laser made of two coupled isospectral optical cavities that support only one transverse mode and several longitudinal ones. We assume that the pumping (electrical or optical) is applied only to one cavity while the other remains passive as illustrated schematically in Fig. 1(a). We denote the identical intrinsic loss coefficients of any mode *n* in either cavity by *α*_*n*_ (which in general can include material and radiation loss as well as any mechanism for extracting laser light such as evanescent coupling to waveguides). Our model so far coincide with that presented in ref. 45. In order to demonstrate the possibility of engineering a more complex lasing selectivity, we depart from ref. 45 (which treats only the case of cavities having equal loss) by assuming that the passive cavity can exhibit an extra contribution to the modal loss and we denote this additional part by *γ*_*n*_. Under these conditions and by eliminating the fast carrier dynamics adiabatically from the rate equations, we arrive at:

1 $$\begin{array}{l}\begin{array}{rcl}i\frac{d{a}_{n}}{dt}-({\omega }_{n}-i{\alpha }_{n}){a}_{n}-i\frac{{g}_{n}}{1+{\sum }_{m=1}^{N}{s}_{nm}{|{a}_{m}|}^{2}}{a}_{n}+{\kappa }_{n}{b}_{n} & = & 0.\\ i\frac{d{b}_{n}}{dt}-({\omega }_{n}-i{\alpha }_{n}-i{\gamma }_{n}){b}_{n}+{\kappa }_{n}{a}_{n} & = & 0.\end{array}\end{array}$$

In equation (1), *a*_*n*_ and *b*_*n*_ are the electric field amplitudes associated with mode *n* in cavities *a* and *b* respectively, and *g*_*n*_ is the gain coefficient in cavity *a* while *κ*_*n*_ is the coupling coefficient between modes *n*. Here, *ω*_*n*_ corresponds to resonance frequency of mode *n*. Finally, *s*_*nn*_ (*s*_*nm*_) are the self (cross) gain saturation coefficient (usually *s*_*nn*_ > *s*_*nm*_)^15^. Below or at the lasing threshold, the eigenvalues of the above system, as expressed in the basis *exp*(−*i*Ω_*n*_ *t*), are given by:

2 $${{\rm{\Omega }}}_{n}^{\pm }={\omega }_{n}+i\frac{{g}_{n}-2{\alpha }_{n}-{\gamma }_{n}}{2}\pm i\sqrt{{(\frac{{g}_{n}+{\gamma }_{n}}{2})}^{2}-{\kappa }_{n}^{2}}$$

Above the lasing threshold, nonlinear interactions are crucial and must be taken into consideration.

Having introduced the model, we now proceed to investigate the robustness and mode selectivity of single longitudinal mode PT symmetric lasers in the following sections. In the rest of this work, single mode operation refers to the longitudinal modes. Additionally, for illustration purpose and in order to gain insight into the physics of the problem, we will consider only two modes per cavity, denoted by *n* = *μ* (the mode corresponds to *ω*_*μ*_ that lies under the peak of the gain curve *g*_*max*_) and the other mode indicated by *ω*_*ν*_ which experiences a gain *rg*_*max*_. As the pump is increased, the value of *g*_*max*_ increases but we assume that the ratio *r* < 1 remains constant. Furthermore, we assumed *κ*_*μ*_ = *κ*_*ν*_ ≡ *κ* ^45^.

## Robustness and stability of single mode PT lasers

**Figure 2:**
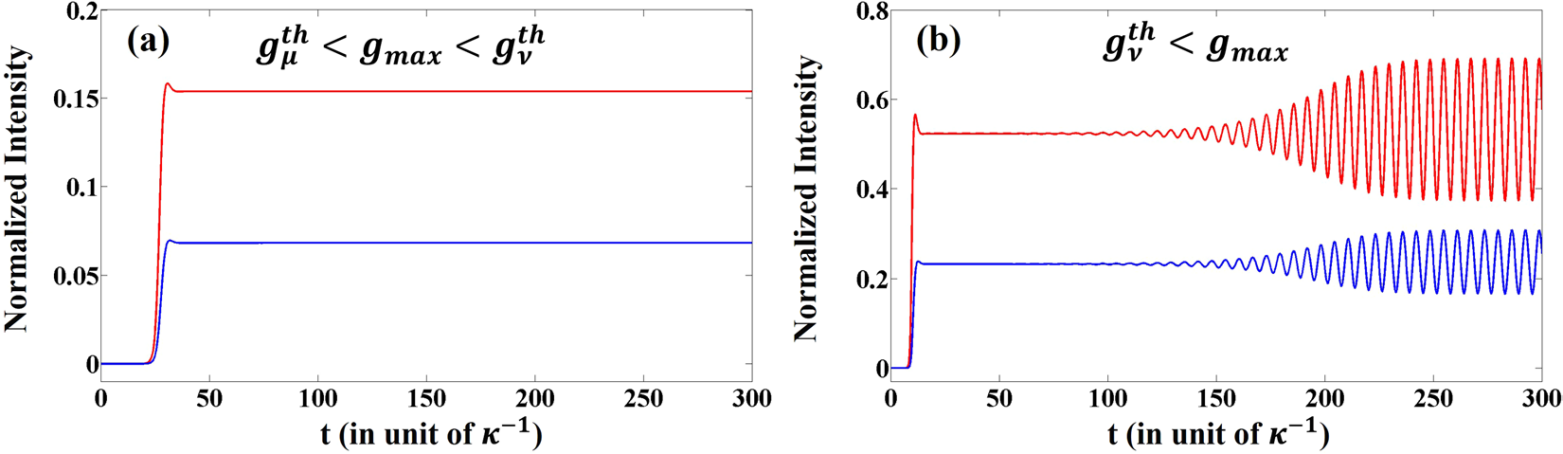
Lasing dynamics of the photonic laser molecule of Fig. 1 in the broken PT regime when (**a**) *g*_*max*_ = 1.0, and (**b**) *g*_*max*_ = 3.3. In both figures, red and blue lines correspond to |*a*_*μ*_ + *a*_*ν*_|^2^ and |*b*_*μ*_ + *b*_*ν*_|^2^, respectively. Here *s*_*μμ*_ = *s*_*νν*_ = 1 and *s*_*μν*_ = *s*_*νμ*_ = 0.25.

In this section, we study the robustness of single mode PT lasers against spectral hole burning effects that might trigger multimode operation. Here we assume that *γ*_*n*_ = 0 and *α*_*μ*_ = *α*_*ν*_ ≡ *α*. According to equation (2), the system starts lasing in the broken PT phase if *α* > *κ*. Under this condition, the lasing threshold is given by ${g}_{\mu }^{th}=\alpha +{\kappa }^{2}/\alpha$ ^49^. To first demonstrate the possible different lasing regimes, we integrate equation (1) numerically for *α* = 1.5, *κ* = 1, *r* = 0.75 and *g*_*max*_ = 1 or *g*_*max*_ = 3.3. This set of parameters are chosen to ensures that the first lasing mode *μ* starts in the broken phase while the other mode is still in the PT phase (equation (2)). Our simulations indicates the existence of two regimes of operation: (I) a single mode steady state emission when ${g}_{\mu }^{th} < {g}_{max} < {g}_{\nu }^{th}$; and (II) a multimode oscillatory behavior when ${g}_{\nu }^{th} < {g}_{max}$, where ${g}_{\mu }^{th}$ is the lasing threshold and ${g}_{\nu }^{th}$ is the second (instability) threshold^1, 2^. Figure 2(a) and (b) depict typical temporal behaviors corresponding to these two regimes, respectively. In plotting the results of Fig. 2 we chose Δ*ω* = *ω*_*μ*_ − *ω*_*ν*_ = 1 for illustration purposes (note that the actual value of Δ*ω* affects only the modal gain ratio *r*). Intuitively, one can understand the behavior observed in Fig. 2 by noting that, in our model, each cavity supports two modes. As the pump levels increase above the first threshold, the first mode starts to lase and both the modal gain ratio *r* and the cross gain saturation act together to suppresses the second lasing mode. However, as the pump is further increased, this steady state solution might become unstable which can then lead to multimode laser oscillations with beating effects.

Further simulations for different system’s parameters shows that ${g}_{\nu }^{th}$ increases as *s*_*μν*_ increases or *r* decreases. These parameters depend on the material system, cavity design (emission frequency and modal overlap), thus one expect different systems to enter the multimode regime at different pumping levels. In order to fully characterize this behavior, we use linear stability analysis. The steady state lasing solutions can be found by substituting: *a*_*μν*_ = *η*_1,3_ *exp*(*iλt*) and *b*_*μν*_ = *η*_2,4_ *exp*(*iλt*) to obtain: *λη*_1–4_ = *f*_1–4_(*η*_1–4_), where the nonlinear functions *f*_1–4_ can be found from equation 1. Here we are interested in the case where only one mode is lasing, i.e. we set *η*_3,4_ = 0, to arrive at:

3 $$\begin{array}{c}M\overrightarrow{\eta }=\lambda \overrightarrow{\eta },\,M=(\begin{array}{ccc}0 & \kappa  & 0\\ \kappa  & 0 & -\alpha \\ 0 & \alpha  & 0\end{array}),\\ \alpha {\eta }_{R1}-\frac{{g}_{{\max }}}{1+{S}_{\mu \mu }{\eta }_{R1}^{2}}{\eta }_{R1}+\kappa {\eta }_{I2}=0\end{array}$$

where, $\overrightarrow{\eta }={[{\eta }_{R1},{\eta }_{R2},{\eta }_{I2}]}^{T}$, with the subscript *T* denoting matrix transpose and the subscripts R, I indicate real and imaginary parts, correspondingly. In writing equation 3 we assumed that *η*_*I*1_ = 0 since only the relative phase between *η*_1,2_ matters. This eigenvalue problem has two different solutions: (1) PT phase: $\lambda =\pm \sqrt{{\kappa }^{2}-{\alpha }^{2}}$, ${\eta }_{1}=\sqrt{(\frac{{g}_{max}}{2\alpha }-1)/{s}_{\mu \mu }}$, ${\eta }_{2}=(\frac{\lambda }{\kappa }+i\frac{\alpha }{\kappa }){\eta }_{1}$ (note that (|*η*_1_| = |*η*_2_|); and (2) Broken PT phase (optimal for laser operation): *λ* = 0, ${\eta }_{1}=\sqrt{(\frac{{g}_{max}}{\alpha +{\kappa }^{2}/\alpha }-1)/{s}_{\mu \mu }}$, ${\eta }_{2}=\frac{i\kappa }{\alpha }{\eta }_{1}$. Note that although these solutions always exist for any pump values, they might not be unique in certain regimes. The stability of these steady state solutions, which determines the lasing characteristics can be found by performing linear stability analysis^2^. By introducing a small perturbation vector $\vec{\delta }q=(\delta {\eta }_{R1 \mbox{-} R4},\delta {\eta }_{I1 \mbox{-} I4})$ over any given solution, we obtain $\dot{\vec{\delta }}q=J\mathrm{.}\vec{\delta }q$ where the Jacobian matrix $J=\frac{\partial ({f}_{1},\ldots ,{f}_{4})}{\partial ({\eta }_{R1},{\eta }_{I1},\ldots ,{\eta }_{R4},{\eta }_{I4})}$ is a function of that particular solution. If *max*(*Re*(*θ*)) > 0, where *θ* are the eigenvalues of the matrix *J*, the perturbation grows and the solution is unstable. On the other hand, the solution is stable if *max*(*Re*(*θ*)) <  0^2^.

**Figure 3:**
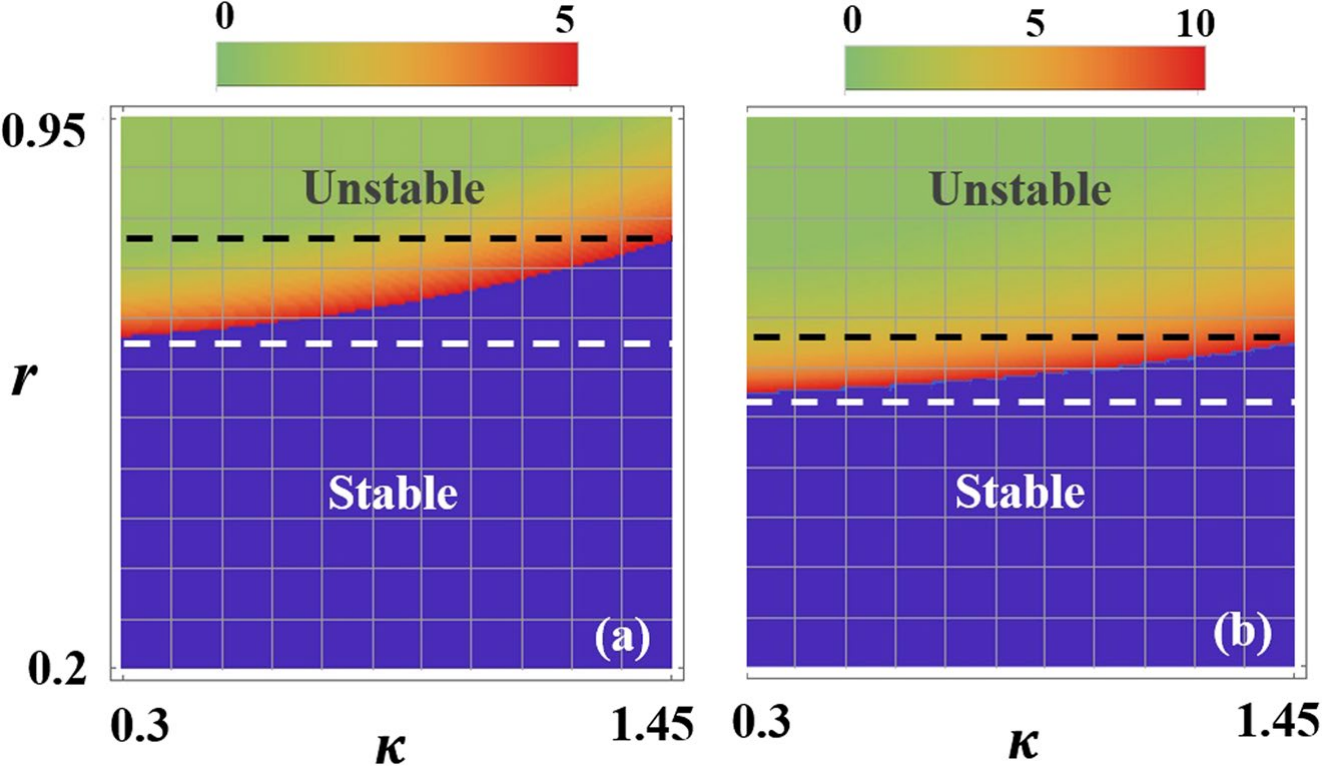
(**a**) Stability maps of a PT symmetric laser as a function of the gain ratio *r* and the coupling coefficients *κ* when *g*_*max*_ = 5. Starting from a low value of *r*, the single mode operation in both cases is found to be stable over a wide range of the coupling parameter *κ* (the regime below the white dashed line). As *r* is further increased, an intermediate domain (between the dashed white and black lines) with mixed stability features is entered. In this regime, the system is unstable for lower *κ* values and can be stabilized by increasing *κ*. Above a certain value for *r*, the laser becomes unstable for all *κ*’s in the specific range. (**b**) Same as in (**a**) but for *g*_*max*_ = 10. Here, as expected, the unstable domain is larger and the values of *r* at which the transitions between the different domains for fixed *κ* is down-shifted. In both figures, the blue shaded areas represent the stable domain and the color map represent the values of ${g}_{\nu }^{th}$.

Figure 3(a) and (b) show that stability maps of a PT symmetric laser when *α* = 1.5, *s*_*μμ*_ = *s*_*νν*_ = 1 and *s*_*μν*_ = *s*_*νμ*_ = 0.5 as a function of the gain ratio *r* and the coupling coefficients *κ* for two different values of the maximum pumping gain *g*_*max*_ = 5, 10. As we can see from Fig. 3(a), starting from a low value of *r* the single mode operation is found to be stable over a wide range of the coupling parameter *κ* (the regime below the white dashed line). As *r* is further increased, an intermediate domain (between the dashed white and black lines) with mixed stability features is entered. In this regime, the system is unstable for lower *κ* values and can be stabilized by increasing *κ*. Above a certain value for *r*, the laser becomes unstable for all *κ*’s in the specific range. Similar behavior is also observed in Fig. 3(b) with the exception that the unstable domain is now expanded at the expense of stable and intermediate domains. Also the values of *r* at which the transitions between the different domains for fixed *κ* is down-shifted.

**Figure 4:**
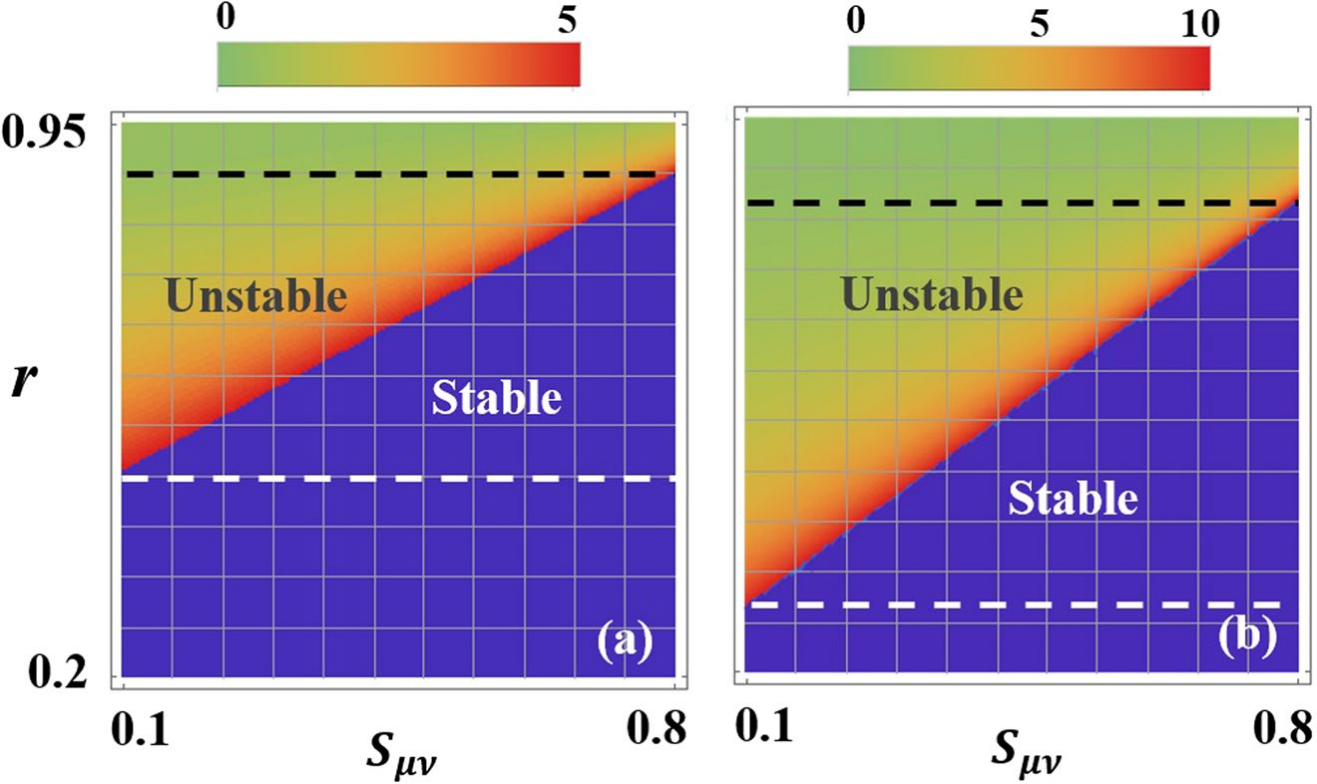
Stability maps as a function of *r* and *s*_*μν*_ for the same parameters used in Fig. 3 when *κ* = 1 and (**a**) *g*_*max*_ = 5 and (**b**) *g*_*max*_ = 10. Here also one can identify three different operating regimes: stable, intermediate and unstable. As discussed in text smaller values for *r* and larger *s*_*μν*_ lead to better stability features.

Figure 4(a) and (b), on the other hand depict the different stability regimes when *κ* = 1 as a function of *r* and *s*_*μν*_ again for different pump values *g*_*max*_ = 5, 10. All other parameters are similar to those in Fig. 3. Here also one can identify three different operating regimes: stable, intermediate and unstable. As expected the area under the unstable domain increases as a function of *g*_*max*_. In perfect agreement with our previous discussion, we find that the system can transit from a stable to an unstable operation as *s*_*μν*_ decreases or as *r* increases.

**Figure 5:**
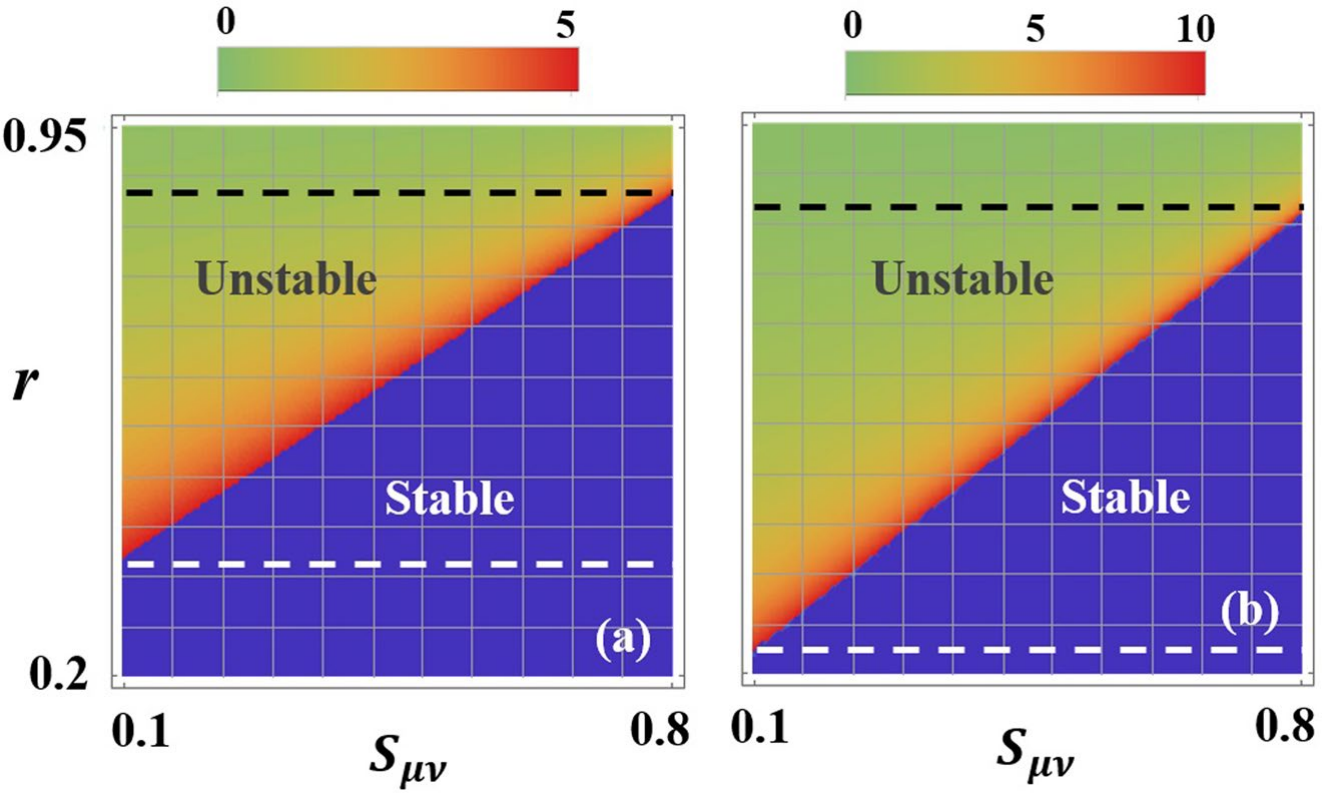
Stability maps for single cavity laser. All parameters are similar to those in Fig. 4. Evidently, in this case, the area under the unstable regime is larger, thus confirming the superior performance of PT symmetric lasers.

Finally, for completeness, we present the stability maps for a single laser cavity (having the same parameters as in Fig. 4) in Fig. 5 where we observe a noticeable expansion of the instability domain. This result indicate that, at least for small *r* values, PT symmetric lasers indeed exhibit superior performance over single cavity systems.

## Mode selectivity via dissipation engineering

In multimode semiconductor laser systems, the lasing thresholds of different modes are determined by the cavity geometry and the central frequency/bandwidth of the gain curve. In the experimental work of ref. 45, the two coupled cavities had identical loss coefficients (i.e. *γ*_*n*_ = 0). As a result, the first lasing mode was the one that falls under the peak of the gain curve (mode *μ* in Fig. 1). Here we explore whether a more general scheme can provide extra flexibility over the mode selectivity. For example let us assume that it is required to engineer the system properties to suppress mode *μ* and promote other mode *ν* to lase first, i.e ${g}_{\nu }^{th} < {g}_{\mu }^{th}$. To do so, we employ a technique similar to that demonstrated in refs 47–49 and, in contrast to the previous section, we assume a finite value for the asymmetric loss coefficients, i.e. *γ*_*μ*,*ν*_ ≠ 0. By neglecting the nonlinear modal interactions for the moment, one can identify four different regimes of operation based on the classification of the lasing modes (either PT or broken PT (BPT)) according to the linear eigenvalues of Eq. (2): (A) PT-PT if (*γ*_*ν*_ + 2*α*_*ν*_)/*r* < *γ*_*μ*_ + 2*α*_*μ*_, (B) PT-BPT if (*γ*_*ν*_ + 2*α*_*ν*_)/*r* < *α*_*μ*_ + *κ* ^2^/(*γ*_*μ*_ + *α*_*μ*_), (C) BPT-PT if (*α*_*ν*_ + *κ* ^2^/(*γ*_*ν*_ + *α*_*ν*_))/*r* < *γ*_*μ*_ + 2*α*_*μ*_, and (D) BPT-BPT if (*α*_*ν*_ + *κ* ^2^/(*γ*_*ν*_ + *α*_*ν*_))/*r* < *α*_*μ*_ + *κ* ^2^/(*γ*_*μ*_ + *α*_*μ*_). These criteria can be experimentally satisfied by using spatial and/or spectral loss distribution as we discuss in more details in the conclusion section.

**Figure 6:**
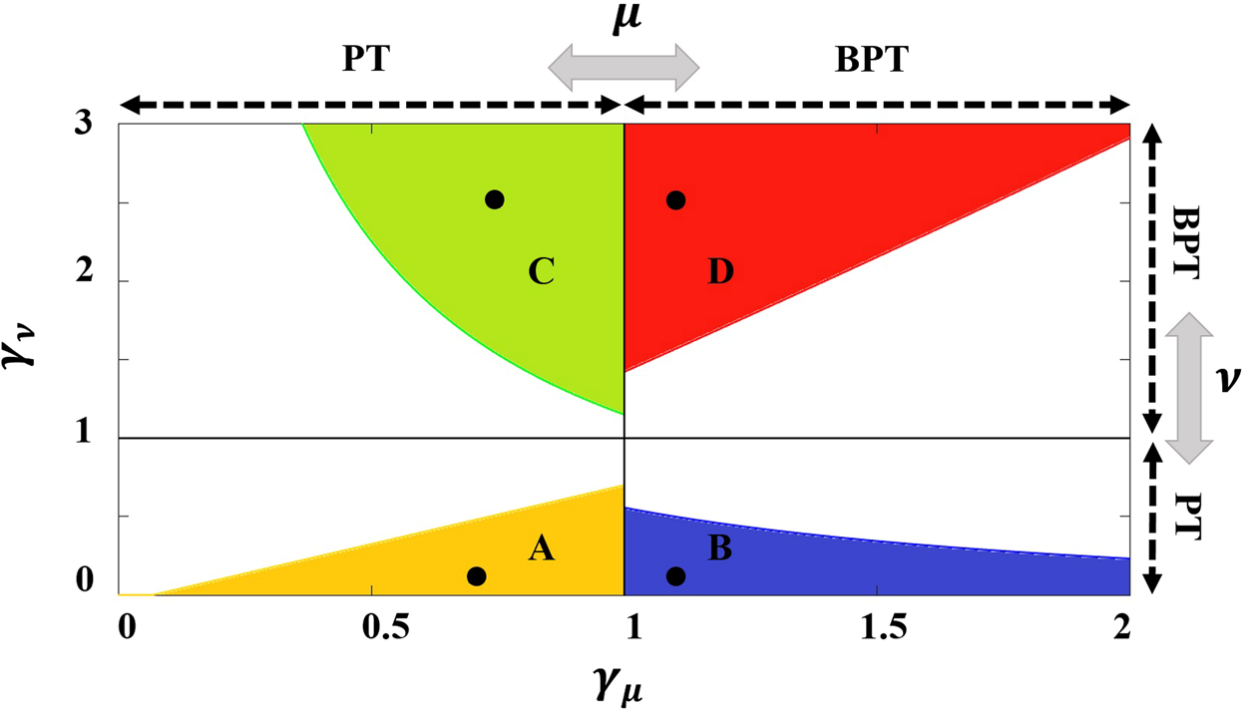
Different lasing regimes for photonic molecule laser as a function of *γ*_*ν*_ and *γ*_*μ*_. The design parameters are chosen to be *κ* = 1, *α*_*ν*_ = *α*_*μ*_ ≡ *α* = 0.1 and *r* = 0.75. The shaded and the white areas correspond to domains where mode *ν* and *μ* lases first, correspondingly.Vertical and horizontal lines mark the transition between PT and BPT phases for modes *μ* and *ν*, respectively. The black dots represents specific design parameters to investigated in more details. Importantly, the different lasing domains are broad enough which allows for large design and implementation tolerance.

**Figure 7:**
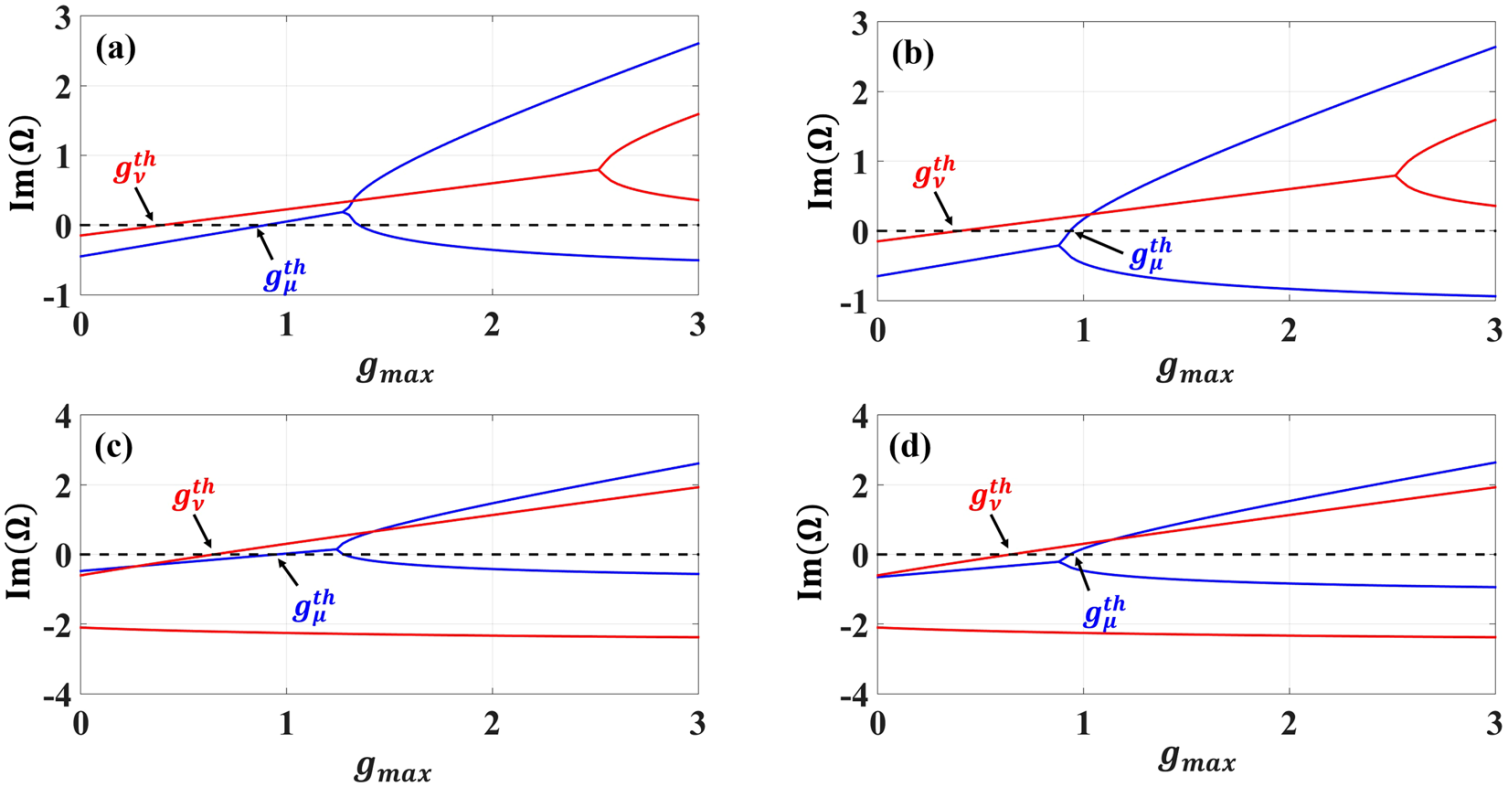
Imaginary part of the eigenvalues of Eq. (2) as a function of *g*_*max*_ are plotted for the different points indicated in each lasing regimes in Fig. 6: (**a**) Both modes lase in PT regime for: *γ*_*ν*_ = 0.1 and *γ*_*μ*_ = 0.7 (PT-PT), (**b**) Mode *ν* lases first in the PT regime followed by mode *μ* lases in broken PT regime for: *γ*_*ν*_ = 0.1 and *γ*_*μ*_ = 1.1 (PT-BPT), (**c**) The first lasing mode *ν* start to lase in broken PT phase then the second mode *μ* in PT regime for: *γ*_*ν*_ = 2.5 and *γ*_*μ*_ = 0.75 (BPT-PT), and (**d**) both modes lase in broken PT phase *γ*_*ν*_ = 2.5 and *γ*_*μ*_ = 1.1 (BPT-BPT). Note that always ${g}_{\nu }^{th} < {g}_{\mu }^{th}$.

As an example, Fig. 6 depicts these domains as a function of *γ*_*ν*,*μ*_ for the parameters mentioned in the caption. We note that although nonlinear interactions play a crucial role in the laser dynamics above threshold, identifying the domains (A)–(D) based on linear analysis is important for choosing a suitable initial design parameters that allow mode *ν* to be the first lasing mode. By inspecting Fig. 6, we can draw several important conclusions. For example, in domain A, *γ*_*μ*_ > *γ*_*ν*_ while the converse is true in C. At first glance, it might appear surprising in this last case that mode *ν* can be still the first lasing mode even when *γ*_*μ*_ < *γ*_*ν*_. However by recalling that in this regime, mode *ν* is in the broken phase while *μ* is in the PT phase, it is straightforward to show that the modal loss of supermode formed by the hybridization of modes *ν* in both cavities is less than that associated with *μ* — a peculiar effect of broken PT phase after crossing exceptional points^44, 49, 54^. In order to further illustrate our results, we plot the imaginary parts of eigenvalues associated with the modes for four different points (each chosen to lie within one of the distinct domains of Fig. 6) as shown in Fig. 7.

**Figure 8:**
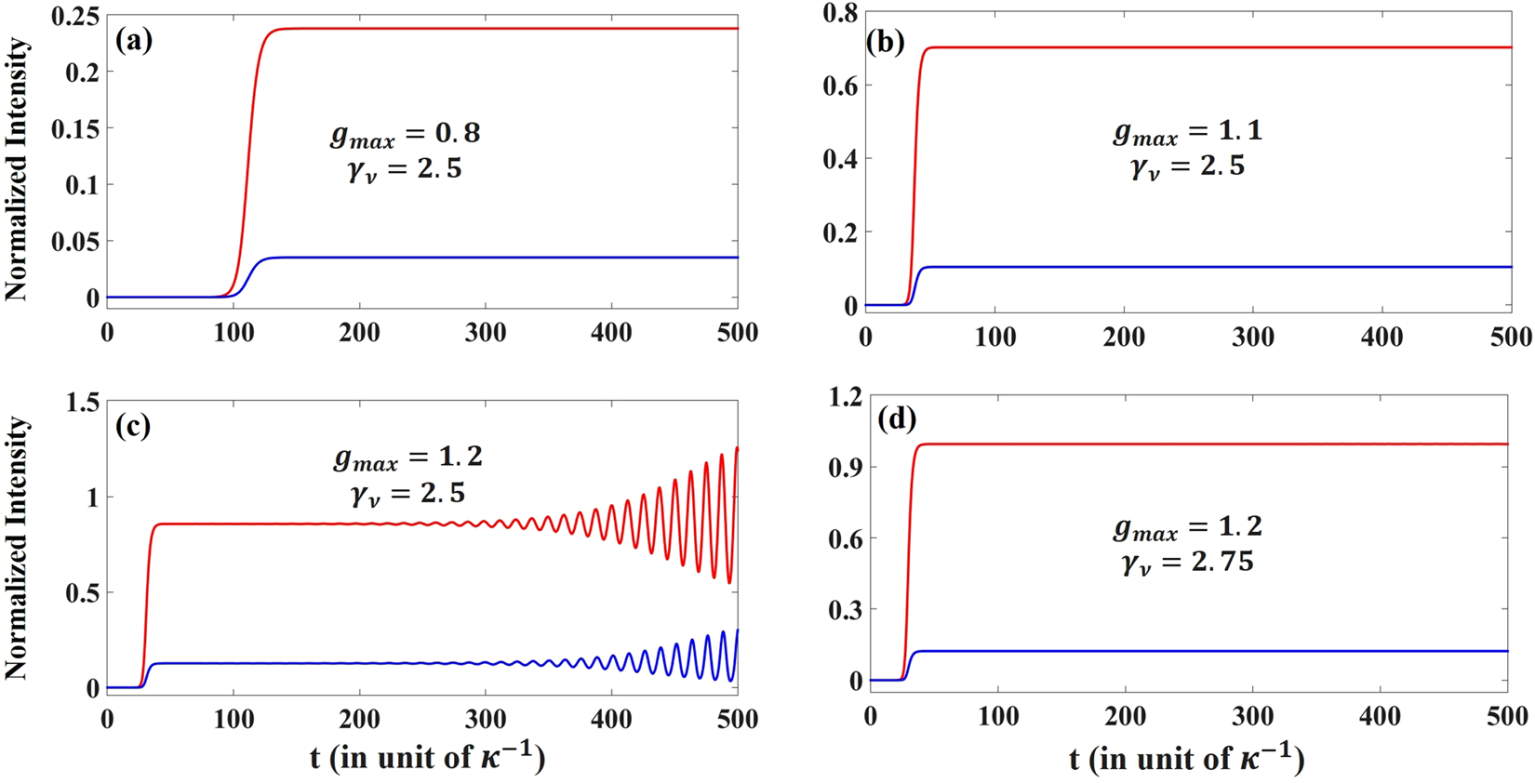
Lasing dynamics for the point shown in domain C of Fig. 6 under different conditions (**a**–**d**). In all simulations, we take *γ*_*μ*_ = 0.75, *α* = 0.1 *κ* = 1, *s*_*μμ*_ = 1 and *s*_*μν*_ = 0.25. Red/blue lines represent the total intensity in the active/passive cavities, respectively. In (**a**,**b**) and (**d**), only mode *ν* is lasing whereas in (**c**) multimode instabilities take place as *g*_*max*_ increases. This instabilities can be mitigated by exploring other values for *γ*_*ν*_ as shown in (**d**).

We have so far discussed the behavior of the linear model described by equation (1) and demonstrated the possibility for mode selectivity in photonic molecule PT lasers by applying pump only to one cavity while engineering the modal dissipation on the second cavity. In that analysis, we neglected nonlinear self gain saturation as well as nonlinear modal interaction via cross gain saturation. Here we study how these nonlinearities affect the lasing dynamics. To do so, we numerically integrate equation (1) for some initial noise. We are particularly interested in domain C where mode *ν* is in the broken phase (thus experiences more gain) while mode *μ* is in the PT phase where it experiences more loss. Figure 8(a) plots the lasing dynamics (total intensity in each cavity) for the point indicated in domain C in Fig. 6. The system reaches its steady state after some transient response. We have inspected the intensity of the individual modes and found that indeed only mode *ν* is participating in the lasing action. In Fig. 8(b) we plot the field intensity under the same conditions as in (a) but for a higher gain value. Again single mode steady state lasing is observed. As the gain is further increased, the lasing action can undergo multimode instabilities manifested by the oscillatory behavior in (c). However, the system can be driven back to its single mode stable operation by adjusting the losses as shown in (d). In practice, for every gain value, a stability map superimposed on Fig. 6 would determine the stable operation regimes.

## Conclusion

In this work, we have investigated two different aspects of PT symmetric lasers: that of the robustness of their single mode operation and the possibility of a more versatile mode selectivity based on generalized loss engineering scheme. We have shown that in general, multimode operation can be triggered by instabilities arising from nonlinear modal interactions. By performing linear stability analysis and presenting the stability map under different conditions, we found that PT symmetry can provide superior performance and robust single mode operation (compared to single cavities) in systems made from microcavities having relatively large FSR. For systems made of long resonators with large optical path between roundtrips, the situation might be different. For example, consider a microring resonator having a radius of *R* = 10 *μm* and a typical waveguide-based edge emitting laser having a length of say *L* = 1 *mm*. Rough estimations based on simplified models gives: $F\equiv FS{R}_{ring}/FS{R}_{strip}=\frac{L}{\pi R} \sim 30$. As a result, the optical modes in edge emitting lasers are more densely packed than those of microrings, resulting in larger *r* values and consequently stronger mode competition which eventually can lead to instabilities (see large *r* regimes in Figs 3 and 4). Future experimental work using these platforms will definitely shed more light on performance of these systems. It will be also interesting to investigate nonlinear laser dynamics in complex laser networks having higher order exceptional points^32^.

Additionally, we have investigated the possibility of controlling the lasing modes by employing a more general scheme of loss engineering. Our analysis shows that by carefully engineering the modal loss in the passive cavity independently from that associated with the active one, a secondary mode can be enhanced at the expense of fundamental mode. This can be achieved for instance by depositing meta-absorbers whose dispersion properties are tailored to match the frequency of the desired mode. The advantage of this approach is the possibility of tuning the absorption spectrum by means of geometric design of the absorbers. However, this can work only if the free spectral range of the cavity is larger than the bandwidth of th meta-absorbers. Another alternative is to use dopant material with narrow absorption bandwidth. However, this is a material-dependent approach and may not provide much flexibility. A different possibility is to exploit the modal intensity distribution to design meta-absorbers that target only certain modes. This strategy has been employed recently in refs 14, 46. This latter approach also opens the door for dynamically controlling the laser emission by using meta-absorbers made of material platforms whose electric properties can be tuned by carrier injection^56^. We explore some of these possibilities in future work.

Finally we note that even though we focused here only on two lasing modes, additional modes (longitudinal or transverse) can be treated exactly in the same manner after extracting their physical parameters (resonant frequencies, quality factors and coupling coefficients) using full electromagnetic simulations. Given that these modes will experience smaller gains, their effect is not expected to alter our results dramatically. In particular, they will change the boundaries between the different lasing regimes without affecting their existence.

## Methods

Our numerical analysis for the linear eigenvalue problem of laser system at threshold was carried out by using standard eigenvalue solver. The dynamics of photonic molecule laser were obtained by solving the nonlinear rate equations using the RK4 algorithm. The linear stability analysis is performed numerically.

## References

[1] Agrawal, G. P. & Dutta, N. K. Semiconductor Lasers, (Springer Science and Business Media, 2013).

[2] Ohtsubo, J. Semiconductor Lasers: Stability, Instability and Chaos, Springer Berlin Heidelberg (2005).

[3] Kapon, E. Semiconductor Lasers I: Fundamentals, (Academic Press, 1999).

[4] Ghafouri-Shiraz, H. Distributed Feedback Laser Diodes and Optical Tunable Filters, (John Wiley and Sons, 2004).

[5] McCall SL, Levi AFJ, Slusher RE, Pearton SJ, Logan RA (1992). Whispering gallery mode microdisk lasers. Appl. Phys. Lett..

[6] Kuwata-Gonokami M (1995). Polymer microdisk and microring lasers. Opt. Lett..

[7] Bayer M (1998). Optical Modes in Photonic Molecules. Phys. Rev. Lett..

[8] Fujita M, Sakai A, Baba T (1999). Ultrasmall and ultralow threshold GaInAsP-InP microdisk injection lasers: design, fabrication, lasing characteristics, and spontaneous emission factor. IEEE Journal of Selected Topics in Quantum Electronics.

[9] Fujita M, Baba T (2002). Microgear laser. Applied Physics Letters.

[10] Nakagawa A, Satoru I, Baba T (2005). Photonic molecule laser composed of GaInAsP microdisks. Applied Physics Letters.

[11] Ishii S, Baba T (2005). Bistable lasing in twin microdisk photonic molecules. Applied Physics Letters.

[12] Li, M. *et al*. Inversed Vernier effect based single-mode laser emission in coupled microdisks. Scientific Reports **5**, Article number: 13682 (2015). 10.1038/srep13682 PMC4557034 26330218

[13] Nozaki K, Nakagawa A, Sano D, Baba T (2003). Ultralow threshold and single-mode lasing in microgear lasers and its fusion with quasi-periodic photonic crystals. IEEE J. Sel. Top. Quantum Electron..

[14] Schlehahn A (2013). Mode selection in electrically driven quantum dot microring cavities. Optics express.

[15] Sorel M (2003). Operating regimes of GaAs-AlGaAs semiconductor ring lasers: experiment and model. IEEE Journal of Quantum Electronics.

[16] Painter O (1999). Two-Dimensional Photonic Band-Gap Defect Mode Laser. Science.

[17] Altug H, Vučković J (2005). Photonic crystal nanocavity array laser. Optics Express.

[18] Khajavikhan M (2012). Thresholdless nanoscale coaxial lasers. Nature.

[19] Bender CM, Boettcher S (1998). Real Spectra in Non-Hermitian Hamiltonians Having PT Symmetry. Phys. Rev. Lett..

[20] Bender CM, Boettcher S, Meisinger P (1999). PT-symmetric quantum mechanics. Journal of Mathematical Physics.

[21] El-Ganainy R, Makris KG, Christodoulides DN, Musslimani ZH (2007). Theory of coupled optical PT-symmetric structures. Optics letter.

[22] Musslimani, Z. H., Makris, K. G., El-Ganainy, R. & Christodoulides, D. N. Optical solitons in PT periodic potentials. *Phys*. *Rev*. *Lett*. **100**, 030402-1-4 (2008). 10.1103/PhysRevLett.100.030402 18232949

[23] Makris, K. G., El-Ganainy, R., Christodoulides, D. N. & Musslimani, Z. H. Beam dynamics in PT-symmetric optical lattices. *Phys*. *Rev*. *Lett*. **100**, 103904-1-4 (2008). 10.1103/PhysRevLett.100.103904 18352189

[24] Guo, A. *et al*. Observation of PT-symmetry breaking in complex optical potentials. Phys. Rev. Lett. **103**, 093902-1-4 (2009). 10.1103/PhysRevLett.103.093902 19792798

[25] Rüter CE (2010). Observation of parity-time symmetry in optics. Nature Phys..

[26] Longhi S (2009). Peschel Bloch Oscillations in Complex Crystals with PT Symmetry. Phys. Rev. Lett..

[27] Chong YD, Ge L, Cao H, Stone AD (2010). Coherent perfect absorbers: time-reversed lasers. Phys. Rev. Lett..

[28] Lin Z (2011). Unidirectional invisibility induced by P T-symmetric periodic structures. Phys. Rev. Lett..

[29] Schindler J, Li A, Zheng MC, Ellis FM, Kottos T (2011). Experimental study of active LRC circuits with PT symmetries. Phys. Rev. A.

[30] El-Ganainy R, Makris KG, Christodoulides DN (2012). Local PT invariance and supersymmetric parametric oscillators. Phys. Rev. A.

[31] Schomerus H (2013). Topologically protected midgap states in complex photonic lattices. Optics Lett..

[32] Teimourpour MH, El-Ganainy R, Eisfeld A, Szameit A, Christodoulides DN (2014). Light transport in PT-invariant photonic structures with hidden symmetries. Physical Review A.

[33] Wiersig J (2014). Enhancing the sensitivity of frequency and energy splitting detection by using exceptional points: application to microcavity sensors for single-particle detection. Phys. Rev. Lett..

[34] Peng B (2014). Parity-time-symmetric whispering-gallery microcavities. Nature Physics.

[35] Jing H (2014). PT-Symmetric Phonon Laser. Phys. Rev. Lett..

[36] Makris KG, Ge L, Türeci HE (2014). Anomalous transient amplification of waves in non-normal photonic media. Phys. Rev. X.

[37] Zhang J (2015). Giant nonlinearity via breaking parity-time symmetry: A route to low-threshold phonon diodes. Phys. Rev. B.

[38] Liu ZP (2016). Metrology with PT-Symmetric Cavities: Enhanced Sensitivity near the PT-Phase Transition. Phys. Rev. Lett..

[39] Monifi F (2016). Optomechanically induced stochastic resonance and chaos transfer between optical fields. Nature Photonics.

[40] Doppler J (2016). Dynamically encircling an exceptional point for asymmetric mode switching. Nature.

[41] Jing H, Özdemir ŞK, Lü H, Nori F (2017). High-order exceptional points in optomechanics. Scientific Reports.

[42] Ramezani H (2017). Non-Hermiticity-induced flat band. Phys. Rev. A.

[43] Jahromi AK (2017). Transparent Perfect Mirror. ACS Photonics.

[44] Liertzer, M. *et al*. Pump-induced exceptional points in lasers. *Phys. Rev. Lett*. **108**, 173901-1-5 (2012). 10.1103/PhysRevLett.108.173901 22680867

[45] Hodaei H, Miri MA, Heinrich M, Christodoulides DN, Khajavikhan M (2014). Parity-time-symmetric microring lasers. Science.

[46] Feng L, Wong ZJ, Ma RM, Wang Y, Zhang X (2014). Single mode laser by parity-time symmetry breaking. Science.

[47] Brandstetter M (2014). Reversing the pump dependence of a laser at an exceptional point. Nat. Commun..

[48] Peng B (2014). Loss-induced suppression and revival of lasing. Science.

[49] El-Ganainy R, Khajavikhan M, Ge L (2014). Exceptional points and lasing self-termination in photonic molecules. Phys. Rev. A.

[50] El-Ganainy R, Khajavikhan M, Christodoulides DN (2015). Supersymmetric laser arrays. Phys. Rev. A.

[51] Teimourpour MH, Ge L, Christodoulides DN, El-Ganainy R (2016). Non-Hermitian engineering of single mode two dimensional laser arrays. Sci. Rep..

[52] Gu Z (2016). Experimental demonstration of PT-symmetric stripe lasers Laser Photon. Rev..

[53] Hassan AU, Hodaei H, Miri MA, Khajavikhan M, Christodoulides DN (2015). Nonlinear reversal of the PT-symmetric phase transition in a system of coupled semiconductor microring resonators. Phys. Rev. A.

[54] Ge L, El-Ganainy R (2016). Nonlinear modal interactions in parity-time (PT) symmetric lasers. Sci. Rep..

[55] Yang J (2017). Nonlinear behaviors in a PDE model for parity-time-symmetric lasers. J. Opt..

[56] Gao Z, Fryslie STM, Thompson BJ, Carney PS, Choquette KD (2017). Parity-time symmetry in coherently coupled vertical cavity laser arrays. Optica.

